# Cross‐Modality Treatment Generalization in Sentence Comprehension in Aphasia: Evidence From Structural Priming and Webcam‐Based Eye‐Tracking

**DOI:** 10.1111/1460-6984.70284

**Published:** 2026-07-12

**Authors:** Willem S. van Boxtel, Peng Zhang, Nadine Martin, Victor Ferreira, Jiyeon Lee

**Affiliations:** ^1^ Department of Speech, Language, and Hearing Sciences Purdue University USA; ^2^ Department of Communication Sciences and Disorders Louisiana State University USA; ^3^ College of Public Health Temple University USA; ^4^ Department of Psychology University of California at San Diego USA

**Keywords:** aphasia, cross‐modality generalization, structural priming, sentence treatments, webcam eyetracking

## Abstract

**Background:**

Acquired neurogenic communication disorders such as aphasia are associated with sentence production and comprehension difficulties. However, sentence‐level treatments for aphasia do not consistently yield generalized gains across modalities, from production to comprehension or vice versa. This limits the effectiveness of current aphasia treatments. In this study, we present structural priming, the phenomenon where repeated grammatical structures are used more frequently and are easier to process, as a potentially effective treatment for sentence‐level deficits in aphasia, and one which may achieve generalization from production to comprehension. Further, we assessed whether lexical overlap between prime and target sentences (lexical boost) affected the strength of cross‐modality generalization.

**Method:**

24 people with aphasia (PWA) and 16 age‐matched controls completed a structural priming sentence production treatment. Their comprehension of trained structures (passives and double‐object datives) was tested with a sentence‐picture matching task before and after training to assess cross‐modality generalization effects. Eye movements were recorded during the comprehension task using webcams.

**Results and Conclusions:**

Response times and fixation times to the target picture were facilitated from pre‐ to post‐training, although comprehension accuracy remained stable. While controls were more facilitated by same‐verb priming, PWA benefited more from different‐verb priming. Improvements in sentence production during training were related to the strength of improvements on the comprehension task across groups. These results suggest that structural priming has the potential to achieve cross‐modality transfer of treatment gains and different‐verb priming may be more effective in aphasia than same‐verb priming.

**WHAT THIS PAPER ADDS:**

*What is already known on the subject*

- Acquired neurogenic communication disorders such as aphasia are associated with sentence production and comprehension impairments. However, sentence‐level treatments for aphasia do not consistently yield generalized gains across modalities, from production to comprehension or vice versa. This severely limits the effectiveness of current treatments.

*What this study adds to existing knowledge*

- In this study, we present structural priming, the phenomenon where repeated grammatical structures are used more frequently and are easier to process after exposure, as an effective treatment for sentence‐level deficits in aphasia, and one which achieves reliable generalization from production to comprehension. Further, we assessed whether lexical overlap between prime and target sentences (lexical boost) affected the strength of cross‐modality generalization. Eye movements were tracked using webcams, which offers a novel and accessible method of online data collection for communication disorders.

*What are the clinical implications of this study?*

- Our results make the case for structural priming as a reliable treatment component for sentence production and comprehension in aphasia, which achieves cross‐modality transfer of treatment gains. Eye movements were reliably recorded through webcams.

## 1 Introduction

Many people with aphasia (PWA) experience difficulty producing and comprehending sentences (Bates et al. 1987; Clark 2011; Thompson 2019). Although the degrees and specific nature of impairments between the production and comprehension modalities might differ, studies suggest that impaired syntactic processing may underlie both sentence production and comprehension difficulties in PWA (Garraffa and Fyndanis 2020; Fahey et al. 2024). Deficits range from impaired message–to–structure and morphological encoding (Cho‐Reyes et al. 2016; Faroqi‐Shah and Thompson 2007) to incomplete or deficient access to syntactic representations, in particular for more complex, resource‐demanding grammatical structures (Schwartz et al. 1994; Berndt et al. 1997; Meyer et al. 2012). Effective treatments for sentence‐level deficits in aphasia, and for sentence comprehension in particular, are nevertheless scarce when compared to other linguistic skills (Schwartz et al. 1994; Kiran et al. 2007; Boyle 2017; Hickin et al. 2022). Moreover, those sentence‐level treatments that do exist show weak, inconsistent transfer of acquired improvements from one modality to the other (Adelt et al. 2018). In particular, while studies have shown some transfer of treatment gains in comprehension transferring to production (e.g., Jacobs and Thompson 2000), transfer of *production* treatment gains to the *comprehension* modality appears especially limited (Poirier et al. 2023). Such cross‐modality transfer is nevertheless crucial if cost‐effective treatments are to be developed. In this study, we focused on production‐to‐comprehension generalization using a *structural priming* treatment paradigm, a recently developed approach for strengthening syntactic processing in aphasia (Lee and Man 2017; Lee et al. 2025; see Lee 2024 for review).

Studies of sentence comprehension in aphasia have benefited from the use of eye‐tracking methodology to gauge online, implicit strategies underlying language behaviors (e.g., Dickey et al. 2007; Hanne et al. 2015; Knilans and DeDe 2015; Lee and Thompson 2011; Lee 2020; for a review, see Sharma et al. 2021). For instance, PWA show reduced predictive gazes to relevant information during real‐time sentence parsing (Hanne et al. 2015), suggesting deficits in the gradual building of syntactic representations in aphasia. Eye‐tracking patterns have also revealed that PWA show delayed and reduced processing of grammatical and morphosyntactic cues (Schumacher et al. 2015), further suggesting impairments to syntactic processing specifically. Crucially, eye‐tracking methods record implicit, time‐sensitive, continuous data which are relatively free from confounds resulting from PWAs’ co‐morbid difficulties such as impaired motor functions (Sharma et al. 2021). Eye‐tracking is therefore a critical tool for assessing fine‐grained, unconscious comprehension patterns.

This paper combines real‐time eye movement recording with an implicit sentence production treatment, aiming to achieve transfer of treatment gains in production to comprehension. Participants in this study completed up to six sessions of structural priming training to improve sentence production. An eye‐tracking‐while‐listening task was administered before and after the production priming training to assess if the participants’ sentence comprehension was improved without direct intervention. We further evaluated whether specific learning conditions underlying structural priming (same v. different‐verb priming) can be manipulated to boost cross‐modality generalization.

### 1.1 Cross‐Modality Generalization for Sentence‐Level Treatments in PWA

Treatments for sentence‐level deficits in PWA are few and far between (Boyle 2017; Kiran et al. 2015; Schwartz et al. 1994). Further, existing treatments are highly situation‐specific and grounded in explicit skills (such as declarative recall of learned information), which can be impaired in PWA (e.g., Martin et al. 2018). Existing treatments for sentence production tend to target either specific real‐life situations, such as Script Training (Goldberg et al. 2012), which trains PWA to explicitly remember scripts to be used in common real‐life situations (e.g., ordering a coffee), or assume that explicitly training specific, complex grammatical structures generalizes to improvements in simpler structures (e.g., Treatment of Underlying Forms; Thompson and Shapiro 2005). Further, treatments for sentence comprehension show variable long‐term maintenance of treatment gains, as well as inconsistent transfer of treatment gains from trained to untrained items and tasks (see Adelt et al. 2018, for a review).

Indeed, studies assessing cross‐modality generalization from sentence production to sentence comprehension, or vice versa, have found inconsistent effects (e.g., Adelt et al. 2018; Schröder et al. 2015; Jacobs and Thompson 2000). The available evidence generally suggests transfer from comprehension to production is more consistent than transfer from production to comprehension. As reported in a meta‐analysis by Poirier et al. (2023) of sentence‐level treatment efficacy in aphasia, a majority of studied individuals across treatment protocols and programs showed some improvements in production measures following comprehension treatment. Conversely, evidence for production‐to‐comprehension generalization is far more disparate, and existing studies (e.g., Weinrich et al. 2001; Harris et al. 2012; Schröder et al. 2015) find inconsistent or weak generalization from the production to the comprehension modality. Indeed, a review of existing sentence‐level treatments by Adelt et al. (2018) found that comprehension‐to‐production generalization occurred for 10 out of 13 included PWA across seven studies; however. out of the 26 included PWA from 12 studies examining generalization in the reverse direction, from production to comprehension, only four showed significant increases in comprehension accuracy. It therefore remains to be seen whether a treatment protocol can be formulated that reliably achieves production‐to‐comprehension generalization.

Achieving cross‐modality generalization has crucial clinical and theoretical importance. Complete absences of such generalization effects have generally been interpreted as supporting distinct, separate cognitive mechanisms underlying sentence production and comprehension. For instance, the widely quoted Bock and Levelt (1994) sentence processing model assumes distinct sentence parsing and speech formulating modules, each operating independently from the other, primarily interacting only by accessing a common mental lexicon (see also Bock et al. 2002). However, several other theoretical models as well as some recent neuropsychological findings have closely associated production and comprehension, positing common levels of psychological representation (e.g., Dickey and Yoo 2010) and some shared neural substrates underlying both production and comprehension (e.g., Matchin et al. 2023; Segaert et al. 2012).

Clinically, past studies report impairment patterns that predominantly affect only one modality in aphasia. For instance, Caramazza and Hillis (1989) detail a participant who performed at ceiling on measures of sentence comprehension, but showed marked impairments on measures of sentence production, including omission of grammatical morphemes and function words. The authors use this participant's patterns to establish the possibility of completely dissociated impairments to production and comprehension (see Mitchum and Berndt 2008, for a review). While some sentence‐level or grammatical functions may therefore rely on shared levels of representation and neural substrates, it is clearly not the case that all facets of the comprehension and production systems overlap. Thus, it remains to be explored whether shared representations could be leveraged to achieve treatment gains across modalities. The efficacy of sentence‐level treatments would be vastly improved if PWA experienced gains in both modalities following training in only one. Such generalized gains would reduce the time commitment required from both patients and clinicians, could ameliorate treatment fatigue in PWA, and ultimately drive down the cost of treatments. Thus, finding a treatment that transcends the behavioral boundaries between both modalities would be of crucial interest to both clinicians as well as to the psychology and neurology of linguistics.

### 1.2 Structural Priming as Cross‐Modality Acquisition

Processing and encoding specific sentence structures causes facilitation of subsequent processing and encoding of the same structure. This effect, known as structural or syntactic priming, is robust in younger adults (Dell and Ferreira 2016; see Pickering and Ferreira 2008 for review), older adults (Lee et al. 2024; Van Boxtel and Lawyer 2023a), individuals with amnesia (Ferreira et al. 2008; Heyselaar et al. 2017), and persons with aphasia (Cho‐Reyes et al. 2016; Saffran and Martin 1997; Zhang and Lee 2025; see Lee 2024 for review). Priming can be detected as increased likelihoods of producing primed structures, facilitated reaction times, attenuated EEG signals, eye movement alterations, and other measures (for a review, see Branigan and Gibb 2017). Crucially, priming effects are cumulative, such that multiple exposures to a syntactic structure increase priming effects (Kaschak et al. 2014, 2011). This could make structural priming highly suitable as a treatment method for sentence processing impairment in aphasia, as the acquisition of structures can be reinforced over time and in multiple treatment sessions (Lee and Man 2017; Rainey et al. 2026).

An extensive body of research has shown that structural priming occurs not only within one modality but also between the production and comprehension modalities, making priming particularly well suited to assessing and effecting cross–modality treatment generalization in aphasia. While structural priming is most frequently attested in production (see Mahowald et al. 2017, for a meta‐analysis), priming effects in comprehension are also reliable, (e.g., Arai et al. 2007; Tooley and Traxler 2010; 2023b, Tooley and Bock 2014). Priming effects from production to comprehension and vice versa are also attested (Branigan et al. 2005; Licofsky and van Hell, 2019). For example, Litcofsky and van Hell (2019) used a bi–directional cross–modality priming paradigm using the active/passive alternation, in which participants first listened to prime sentences before describing pictures, or vice versa. Comprehension‐to‐production priming was found as participants produced more passive descriptions following reading of passive sentences. Critically, however, EEG signals during reading of passives showed reduced N400 amplitudes on critical regions following production of passive sentences, evidencing production‐to‐comprehension priming. These results are particularly salient given that the same participants showed both directions of generalization, suggesting that priming taps levels of representation accessible to both language production and comprehension. Litcofsky and van Hell's (2019) findings may also align with studies reporting shared neural representations for some production and comprehension tasks: for instance, Segaert et al. (2012) conducted within‐modality and cross‐modality structural priming experiments using auditory repetition and picture description, while functional magnetic resonance images (fMRI) were obtained. Despite the different modalities and task demands underlying each experiment, the same brain regions showed fluctuations in activity, suggesting that structural priming activates resources and functions which are shared between production and comprehension.

People with aphasia also exhibit structural priming effects, adding to the potential utility of priming as an effective treatment (Lee 2024). PWA produce primed structures more frequently following exposure to that structure (Cho‐Reyes et al. 2016; Saffran and Martin 1997). Priming‐based facilitation in sentence comprehension was also reported in PWA (Lee et al. 2019a; Keen and Lee 2024). Structural priming has even been applied to longer–term rehabilitation protocols, where effective and long–lasting sentence production improvements have resulted (Lee and Man 2017; Rainey et al. 2026; Lee, Man et al. 2019; Lee et al. 2025). For instance, Lee et al. (2025) trained PWA and age‐matched controls to produce grammatically complex structures (passives and double‐object datives) using a structural priming paradigm, administered in three training sessions separated by at least a day each. During priming training, participants heard and then read prime sentences and completed target sentence production. Importantly, participants completed a set of sentence production probes before and after training. Lee et al. not only found significant improvements to PWAs’ sentence production following training, but also that these improvements were maintained a week after training ceased. These results are highly promising for the adoption of structural priming as a formal treatment component for aphasia.

Some evidence further suggests that structural priming could facilitate access of syntactic information between modalities in PWA. Man et al. (2019) used a scripted dialogue‐like priming task, where PWA and the experimenter took turns describing pictures with the goal of identifying matching pictures. The authors found that PWA produced more passive sentences after hearing their interlocutor (experimenter) produce passives as compared to actives, indicating evidence for comprehension ‐to‐production priming (see also Van Boxtel et al. 2023; Lee et al. 2023; Lee, Man et al. 2019b). Keen and Lee (2024) further demonstrated production–to–comprehension priming in a sentence‐picture matching task. Their targeted sentences comprised those with syntactically ambiguous prepositional phrases (e.g., “The teacher is poking the monk with a bat”), where a high attachment interpretation involves the teacher having the bat, and a low–attachment interpretation implies the monk having the bat. Keen and Lee (2024)’s participants completed priming blocks in which they produced sentences with unambiguous low attachment phrases. In comprehension post‐tests using a sentence–picture matching task, participants were free to choose whichever attachment they preferred. Both controls and PWA showed persistent, reliable preferences for the attachment type with which they were primed to produce during the priming block, evidencing robust production‐to‐comprehension priming effects.

### 1.3 Abstract Syntactic Priming and the Lexical Boost

While a growing number of studies support the clinical utility of structural priming treatments in PWA, which learning conditions are most essential to long‐lasting gains is still being investigated (see Lee 2024 for review; Lee et al. 2025; Van Boxtel and Miller 2025). Different learning mechanisms are thought to underpin structural priming (e.g., Chang et al. 2006; Reitter et al. 2011; Heyselaar et al. 2021; Pickering and Garrod 2004), which could have important implications for using priming as a treatment. Lee et al. (2025), for instance, found that it is increased opportunity to activate the target syntactic structure, rather than processing of alternating syntactic structures, that created more long‐term effects in PWA. A group of PWA and controls received three sessions of priming training. Lee et al. (2025) found that alternating‐structure priming (in which primes include both the target passive and its syntactic alternative, the active) resulted in weaker priming effects in PWA than single‐structure priming, in which only passives were presented. The opposite pattern was found in controls, who benefited more from alternating‐structure priming, which Lee et al. hypothesize to be the result of error‐based learning. This finding highlights that priming effects and enhancements found in healthy speakers may not necessarily yield the same effects in PWA. Among the most well‐established amplifications of priming is the lexical boost: when lexical material (usually, a lexical head such as a main verb) overlaps between prime and target sentences, priming effects are larger compared to when there is no repeated lexical information between prime and target sentences (e.g., Hartsuiker et al. 2008; Tooley 2020; Pickering & Branigan 1998). However, lexical boost effects are not consistently observable across modalities (Segaert et al. 2013; Traxler et al. 2014) and may present problems for language‐impaired populations (e.g., Man et al. 2019). Determining what learning conditions, result in the most optimal treatment parameters for aphasia is therefore critical.

Abstract syntactic priming, in the absence of lexical overlap, taps syntactic representations without relying on lexical information or explicit memory: this is evidenced by the well‐established phenomenon that priming occurs even if no lexical material is repeated between prime and target, and without conscious speaker awareness (Hartsuiker et al. 2008; Bock 1986). Abstract priming is therefore highly implicit (Chang et al. 2012), and a large body of evidence suggests implicit *learning* is the primary mechanism underlying abstract syntactic priming (e.g., Heyselaar et al. 2021). Indeed, cumulative abstract priming, in which priming effects become greater with more exposure to a prime, also implies implicit learning (Kaschak et al. 2011, 2014). On the other hand, lexically‐mediated priming may result in stronger effects compared to abstract priming alone because of possible additive priming on both lexical and syntactic levels (Hartsuiker et al. 2008; Pickering and Branigan 1998; Traxler et al. 2014). While the strength of priming‐induced training effects may therefore be amplified if lexical overlap is introduced during training, it is unclear whether cognitive‐linguistic deficits associated with aphasia will prevent lexical boost effects from occurring (Potagas et al. 2011; Martin et al. 2018). Indeed, existing results from lexical amplification of priming in aphasia are inconsistent, with some studies finding intact lexical enhancement of priming effects (e.g., Yan et al. 2018; Lee et al., 2023), but others yielding null results (e.g., Van Boxtel et al. 2023; Man et al. 2019; for a meta‐analysis, see Van Boxtel and Miller, 2025). Further, whether lexical boost effects generalize across modalities in PWA is unknown.

In sum, past findings of cross‐modality treatment generalization in aphasia report an inconsistent pattern, and while structural priming in healthy and impaired groups may transfer across modalities, it is unknown whether PWA show systematic improvements to both modalities following priming training in one modality. Further, the learning conditions which may modulate or enhance cross‐modality generalization remain largely unexplored. Thus, this study focuses on investigating long‐lasting cross‐modality generalization effects in comprehension following structural priming training in production while manipulating lexical overlap between primes and targets.

### 1.4 The Present Study

**FIGURE 1:**
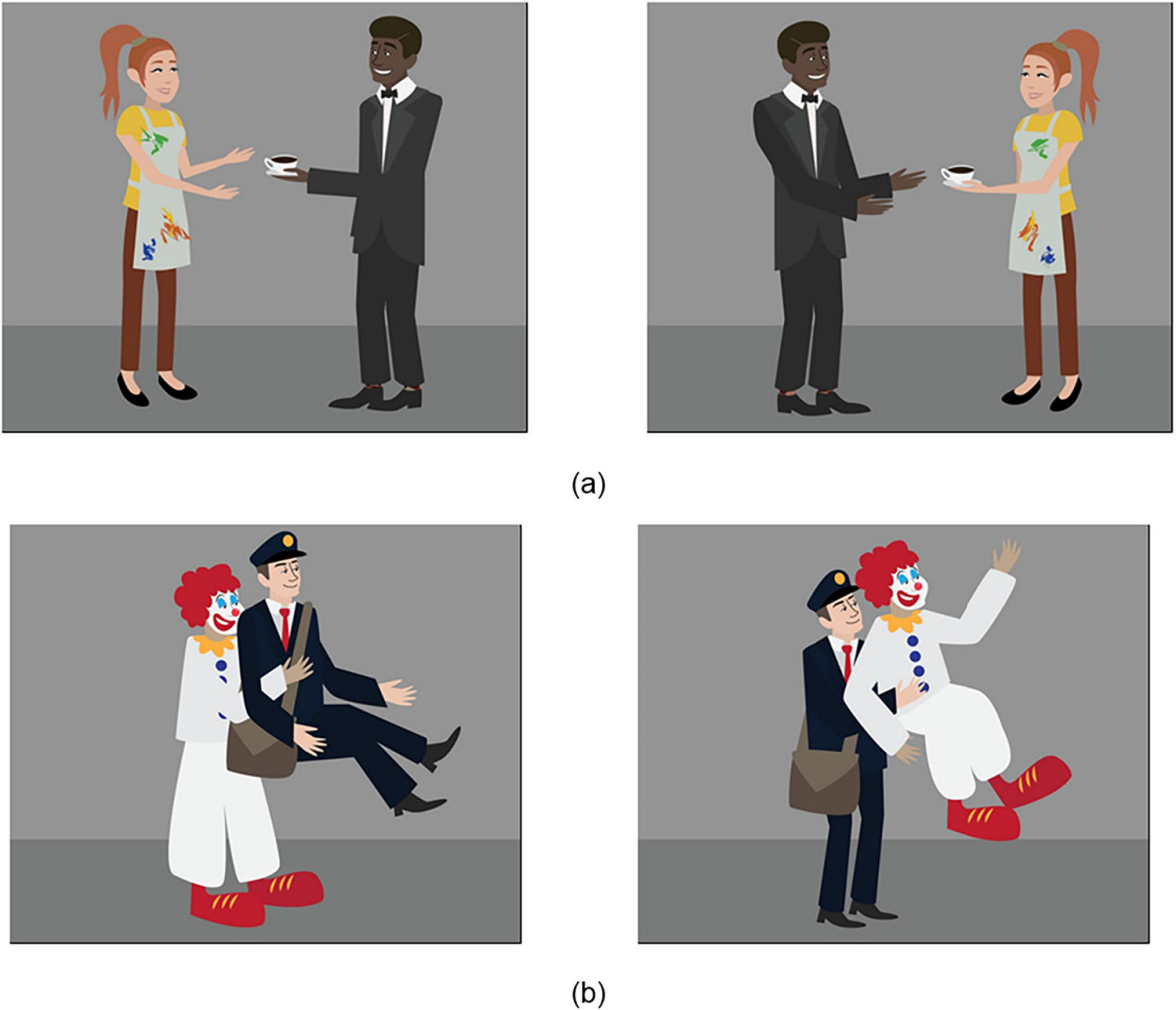
Example sentence comprehension probe stimuli for passive wh‐questions (Figure 1a, target “Who is lifted by the clown here?”), and double‐object dative wh‐questions (Figure 1b, target “Who is giving the groom the coffee?”), administered at baseline and post‐tests.

The purpose of the current study was to examine the effects of structural priming production training on comprehension of sentences in PWA and controls using an eyetracking task in an effort to assess whether structural priming leads to cross‐modality generalization in aphasia. We used a baseline‐training‐post‐testing design, in which participants were tested before training, one day after training ceased (immediate post‐test, or P1), and one week after P1 (delayed post‐test, or P2). Participants completed up to six structural priming training sessions (3 sessions per target sentence type) between baseline and P1, in which they heard and then orally read prime sentences, including passive (e.g., “The clown is lifted by the pilot”) and double‐object dative (e.g., “The artist is giving the groom the coffee”) structures. Participants were trained either using same‐verb priming, in which two primes  with the same main verb were read, or with different‐verb priming, in which both primes contained a different main verb. To assess cross‐modality generalization from sentence production training to sentence comprehension, participants completed an auditory sentence–picture matching task while eye movements were recorded using webcams before and after training. The comprehension task included passive and double‐object wh‐questions for experimental trials, as well as locative wh‐questions for control trials. Each trial presented a target picture as well as a foil in which thematic roles were reversed (see Figure 1 for examples). Our dependent variables included accuracy and response times on the sentence‐picture matching task, as well as proportions of gazes to either the target or foil picture. Main independent variables included group (control v. PWA), session (baseline v. P1 v. P2), and verb overlap condition (same v. different‐verb priming training).

Specifically, we asked three questions with associated hypotheses

(1) Does structural priming training in sentence production generalize to sentence comprehension in PWA?

‡. Hypothesis: if structural priming training improves syntactic processing central to both modalities, participants will show improved comprehension accuracies, faster response times, and increased fixations to the correct target pictures during eyetracking after the training compared to baseline.

(2) Is the strength of cross‐modality generalization affected by performance on measures of sentence production?

‡. Priming‐induced improvements to sentence production should be directly related to, and predictive of, improvements in sentence comprehension.

(3) Which training condition (same verb vs. different verb) results in greater cross‐modality generalization effects in controls and PWA?

‡. If lexical overlap enhances treatment gains, both groups will show higher accuracies, faster response times, and increased target fixations following same‐verb compared to different‐verb priming. If no lexical boost effects are observed, null effects of training condition were predicted.

## 2 Methods

### 2.1 Participants

Twenty‐four persons with aphasia (PWA) and 16 healthy controls participated in this study. Control participants were included for two main reasons: (a) to further validate the webcam‐based eye‐tracking method used in this study; (b) to examine whether conditional differences between same‐verb and different‐verb priming are generalizable across individuals or specific to aphasia. Both samples were identical to the samples reported in Van Boxtel et al. (2025). Participants provided informed consent and were compensated for their participation. This study received full approval from the local institutional review board and is listed on ClinicalTrials.org (NCT05415501). Groups were matched for age (M_PWA_ = 59.2, SD = 11.4; M_Control_ = 63.4, SD = 11.1; *t* = − .988, *p* > .05) and years spent in formal education (M_PWA_ = 16.3, SD = 1.79; M_Control_ = 16.7, SD = 1.35; *t* = ‐.722, *p* > .05). Control participants further confirmed they had not been diagnosed with language or cognitive impairments, were generally healthy, and were native monolingual speakers of North American English. The Cognitive‐Linguistic Quick Test Plus (CLQT+; Helm‐Estabrooks 2017) was administered to control participants to screen for cognitive impairments. Only participants who scored within normal limits on this screening tool were included. Two additional control participants who did not meet inclusion criteria were excluded from the study. All control participants took part in the study in‐person in the laboratory, while all PWA but one took part remotely. Two PWA faced technological difficulties during one of their post‐testing sessions, leading to missing data for those sessions. Procedures and recordings were exactly the same for in‐person and remote participants (see Section 2.4 for procedure details).

All PWA were diagnosed with aphasia resulting from a left‐hemisphere stroke, with at least 6 months intervening between stroke onset and study participation. PWA were 74.7 months post‐stroke onset on average (SD = 66.7; [16, 238]). All PWA were assessed with a set of standardized clinical tests to determine the presence and severity of aphasia, including the Western Aphasia Battery—Revised (WAB‐R; Kertesz 2007), the Northwestern Assessment of Verbs and Sentences (NAVS; Cho‐Reyes and Thompson 2012), portions of the Comprehensive Aphasia Test (CAT; Swinburn et al. 2004), and the Philadelphia Comprehension Battery (PCB; Saffran et al. 1988). See Table 1 for relevant PWA testing scores on subtests of the WAB‐R, NAVS and PCB. Comprehension and production abilities varied across participants, but all PWA demonstrated relatively preserved ability to produce and comprehend single words and some simple sentences. Inclusion criteria for PWA were as follows: Auditory Comprehension scores on the WAB–R of 6/10 or higher, a total score of 70% or higher on the Spoken Word Picture Matching test of the PALPA, a score of 50% or higher on the Verb Naming Test of the NAVS, and a score of at least 30% on the Argument Structure Production Test of the NAVS. Finally, we administered the Symbol Cancellation and Design Memory subtests of the CLQT+ to PWA to screen for attention and memory deficits using non‐verbal tasks. All PWA scored at least 8/12 and 4/6 on those subtests, respectively.

**TABLE 1:** Language testing scores for PWA participants, with means (*μ*) and standard deviations (*σ* ^2^) included. Abbreviations: NHL = non‐Hispanic/Latino; W = white; HL = Hispanic/Latino; DND = Did Not Disclose; B/AA = Black/African American; NH/PI = Native Hawaiian/Pacific Islander; WAB‐R = Western Aphasia Battery‐Revised; Rep = Repetition; AC = Auditory Comprehension; AQ = Aphasia Quotient; NAVS = Northwestern Assessment of Verbs and Sentences; VNT = Verb Naming Test; VCT = Verb Comprehension Test; ASPT = Argument Structure Production Test; SPPT = Sentence Production Priming Test; SCT = Sentence Comprehension Test; CAT = Comprehensive Aphasia Test; PCB = Philadelphia Comprehension Battery.

No	Age	Education (Years)	Months post‐onset	Ethnicity	Race	WAB‐R					NAVS					CAT	PCB		
						Naming	Fluency	Rep	AC	AQ	VNT	VCT	ASPT	SPPT	SCT	Compr of sentences %	Reversible	Lexical	Total
1	31	16	84	NHL	W	8.8	9	10	10	93.6	81.8	100	87.5	93.3	93.3	69	57	100	97
2	43	16	29	NHL	W	8.8	6	7.6	6.4	73.6	95.5	100	96.9	76.7	93.3	94	100	100	78
3	64	14	63	NHL	W	9.0	9	9.4	10	92.8	77.3	100	100	96.7	100	56	80	97	100
4	57	16	34	NHL	W	8.1	6	9.4	9.9	82.7	81.8	95.5	96.9	93.3	100	75	93.3	700	100
5	65	16	32	NHL	W	8.9	9	10	10	91.7	81.8	100	100	96.7	100	81	100	100	98
6	79	18	106	NHL	W	6.5	4	7.7	8.5	65.3	40.9	100	56.3	16.7	96.7	81	90	96.6	88
7	60	18	48	NHL	W	8.6	6	8.2	8.9	81.4	59.1	100	84.4	56.7	66.7	94	96.6	100	88
8	58	18	25	HL	HL	7.8	5	8.7	9.6	78.1	50.0	100	87.5	76.7	96.7	69	76.7	100	93
9	58	12	59	NHL	B/AA	7.9	6	6.9	9.7	78.9	81.8	100	87.5	76.7	86.7	81	80	100	90
10	53	16	75	NHL	W	9.2	6	6.8	7.9	77.7	100	100	93.8	60.0	73.3	81	79.3	96.6	88
11	69	12	41	NHL	W	4.4	8	5.9	6.5	63.5	50.0	81.8	81.3	3.3	66.7	94	50	76.6	63
12	81	12	42	NHL	W	5.1	5	6.2	8.0	64.5	54.6	90.9	84.4	16.7	70.0	63	73.3	86.6	80
13	75	18	16	NHL	W	7.8	4	9.1	9.0	75.7	63.6	95.5	93.8	86.7	93.3	94	80	96.6	88
14	59	16	51	NHL	W	9.4	9	8.8	9.8	92.0	90.9	100	100	90.0	93.3	88	93.3	96.6	95
15	66	15	40	NHL	W	8.8	9	9.9	9.9	93.2	90.9	100	100	90.0	100	75	93.3	96.6	95
16	58	18	303	NHL	W	7.8	6	8.4	8.0	76.3	63.6	90.9	78.1	16.7	66.7	69	47	93.3	70
17	39	15	67	NHL	NH/PI	7.9	5	7.3	7.8	72.0	72.7	100	87.5	40.0	70.0	50	60	93.3	77
18	53	16	92	NHL	W	8.8	6	7.1	7.4	74.6	72.7	90.9	78.1	63.3	90.0	69	63.3	80	72
19	63	15	60	NHL	W	9.1	6	8.7	10	85.6	95.5	100	96.9	96.7	93.3	94	93.3	96.6	95
20	58	18	61	NHL	B/AA	8.9	4	5.6	8.5	69.9	81.8	100	81.3	3.3	53.3	69	63.3	86.6	72
21	60	18	94	NHL	W	8.5	6	5.6	7.6	71.4	86.4	95.5	90.6	10.0	43.3	81	60	83.3	75
22	49	18	114	NHL	W	9.2	6	9.6	9.8	87.1	100	100	93.8	96.7	100	100	100	100	100
23	61	16	19	DND	DND	7.5	6	7.3	9.8	79.2	86.4	100	93.8	60.0	93.3	81	86.67	100	93
24	62	18	238	NHL	W	8.1	6	9.1	8.3	78.9	45.5	95.5	87.5	70.0	76.7	81	60	96.7	78
*μ*	59.2	14.9	74.7			8.1	6.3	8.1	8.8	79.2	75.2	97.3	89.1	62.0	84.0	78.7	70.5	90.6	86.4
SD	11.4	3.9	66.7			1.2	1.6	1.4	1.2	9.2	17.8	4.6	9.9	33.6	16.4	12.9	26.9	20.3	11.0

### 2.2 Materials

This study comprised baseline testing, training sessions, and post–testing. Materials for this study, therefore, included production and comprehension stimuli administered at baseline and post–testing and the stimuli used during the priming training sessions. We discuss each set of materials in turn.

#### 2.2.1 Comprehension Task Stimuli

Target structures for the production training included passives (Theme–Verb–Agent, e.g., “The clown is lifted by the mailman”) and double‐object datives (Agent–Verb–Goal–Theme, e.g., “The artist is giving the groom the coffee”), as previous studies have shown these sentences are difficult for PWA to produce and comprehend (e.g., Cho‐Reyes et al. 2016; Man et al. 2019; Meyer et al. 2012). Therefore, for the sentence comprehension probe task, target stimuli included passive wh‐questions (“Who is lifted by the clown here?”) and double‐object (DO) wh‐questions (“Who is giving the groom the coffee?”). Figure 1 illustrates target and foil images for each target sentence type in the comprehension task. For each question, a target picture and a foil picture were presented, depicting reversed thematic roles of the animate characters, as exemplified in Figure 1. Participants were asked to select a correct picture in response to the wh‐question that they heard. In addition, we included wh‐questions with padded locatives (e.g., “Which cat is on the log next to the river?”) as control stimuli. We included these control stimuli to assess whether observed pre‐ to post‐testing improvements on target wh‐questions were in fact the result of our structural priming manipulation rather than of repeated exposure of the sentences. Padded locatives were selected as control stimuli because while the processing of padded modifiers is expected to be challenging for PWA, they are linguistically different from our target wh‐questions in that they do not require understanding of thematic roles of two animate characters. All sentences were audio recorded by a female native speaker of English and were normalized at 70 dB for volume.

The sentence comprehension task comprised 100 trials of which 60 were experimental trials, equally subdivided into 20 passive, 20 DO, and 20 locative wh‐questions. The remaining 40 trials were fillers, which comprised intransitive declarative wh‐questions (e.g., “Which man is cleaning?”). In locatives, nouns were used a maximum of five times each, while in datives and passives each critical noun (agent and theme for passives; agent and goal for DO datives) appeared only once in each sentence position (N1 or N2). Each verb was repeated twice in both DOs and passives, but with different nouns, creating all unique sentences. Word length (in syllables) and frequency (extracted from the SUBTLEX database, see Brysbaert et al. 2012) of nouns and verbs were matched between structures. There were no differences in length or frequency of the noun included in critical stimuli between passives and DO trials (length *t*(37.7) = .515, *p* > .05; frequency *t*(37.7) = ‐1.001, *p* > .05). In addition, verbs were equally long between passive and DO trials (*t*(19) = 1.453, *p* > .05) but DO verbs were more frequent than passive verbs (*t*(33.6) = 6.258, *p* < .001). In our locative control stimuli, there were no differences in length or frequency between the first and second included nouns (“cat” and “log”; length *t*(37.7) = .370, *p* > .05; frequency *t*(32.2) = 1.222, *p* > .05), or between the second and third nouns (“log” and “river”; length *t*(31.5) = ‐.853; *p* > .05; frequency *t*(36.9) = ‐1.839, *p* > .05), or between the first and third nouns (“cat” and “river”; length *t*(33) = ‐.556, *p* > .05; frequency *t*(30) = ‐.988, *p* > .05). For a full list of stimuli, see our online Supplementary Materials at https://osf.io/rhbue/.

#### 2.2.2 Training Session and Sentence Production Probe Stimuli

**FIGURE 2:**
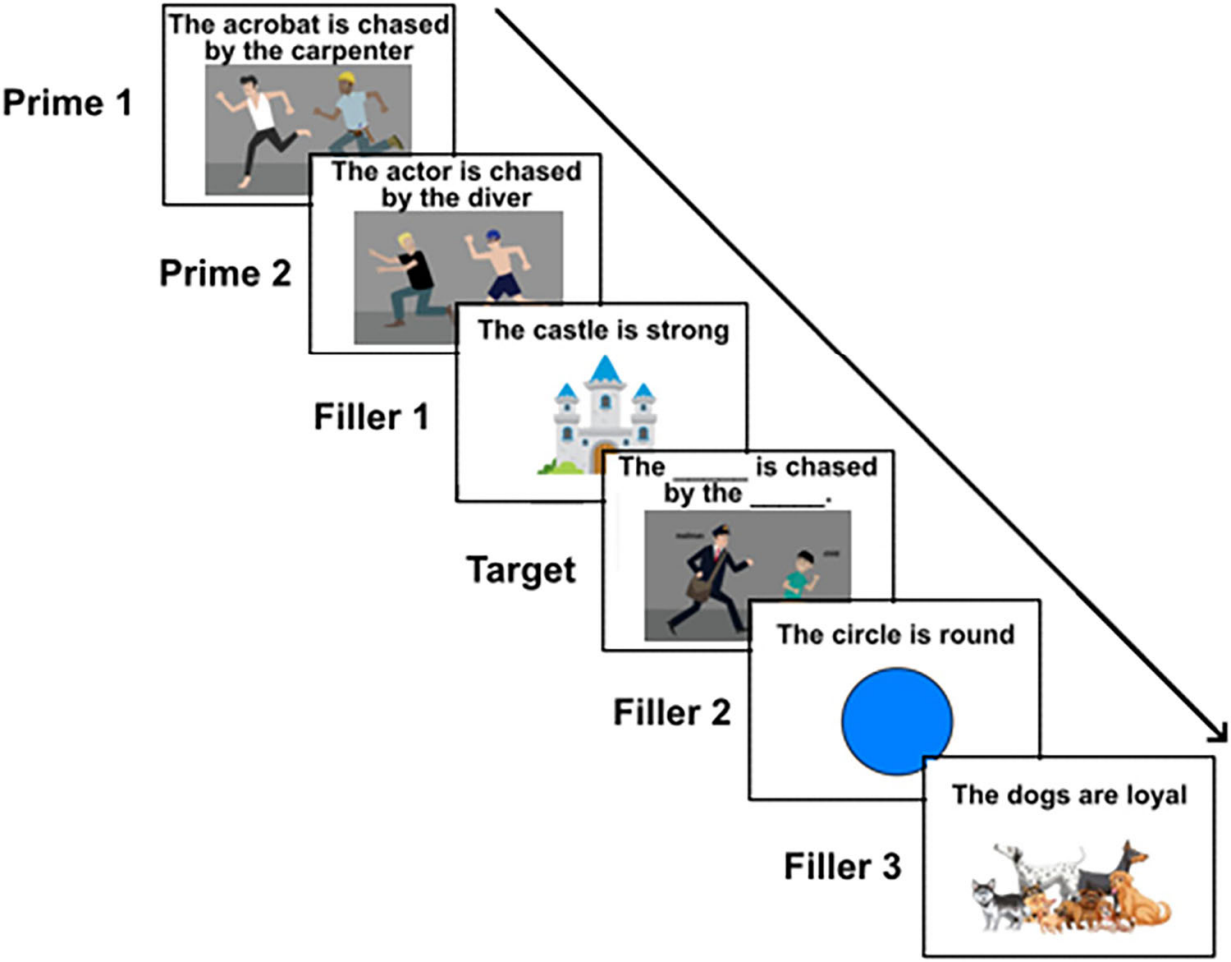
Example training trial sequence for a passive, same‐verb trial. Two primes preceded one filler, followed by the target sentence completion item. This is followed by two more fillers before the next trial commences.

Training sessions comprised a structural priming paradigm in which participants heard and orally read prime sentences. Participants completed either three or six of these sessions depending on eligibility, and passive and DO sentences were trained in separate sessions (see section 2.3). Each training session comprised forty trials, each trial consisting of two prime sentences, one filler sentence, a target sentence completion, and additional two filler sentences (see Figure 2). Primes and targets were built using 80 action‐describing pictures and sentences (40 DOs, 40 transitives). Ten dative and 10 transitive verbs were prepared and each verb was repeated twice as target and twice as prime, paired with different nouns in each repetition, thus, creating 80 unique sentences. All nouns were animate, human characters, except theme nouns in dative items (e.g.,“coffee”). Semantic overlap between nouns within a single training trial was avoided. The position of the agent character in the pictures was counterbalanced such that the agent appeared on the left‐hand side of the picture in half of sentences, and on the right‐hand side in the other half. More details on stimuli development can be found in Lee et al. (2025).

Two training tasks identical in structure were built to evaluate two experimental conditions: in the same‐verb condition, both primes and the subsequent target shared a main verb (see Figure 2 for an example). Trials in the different‐verb condition consisted of primes that did not share a lexical verb with the target (e.g., *push, kiss, chase*). All controls and most PWA received both training conditions in counterbalanced order (see Section 2.4).

**FIGURE 3:**
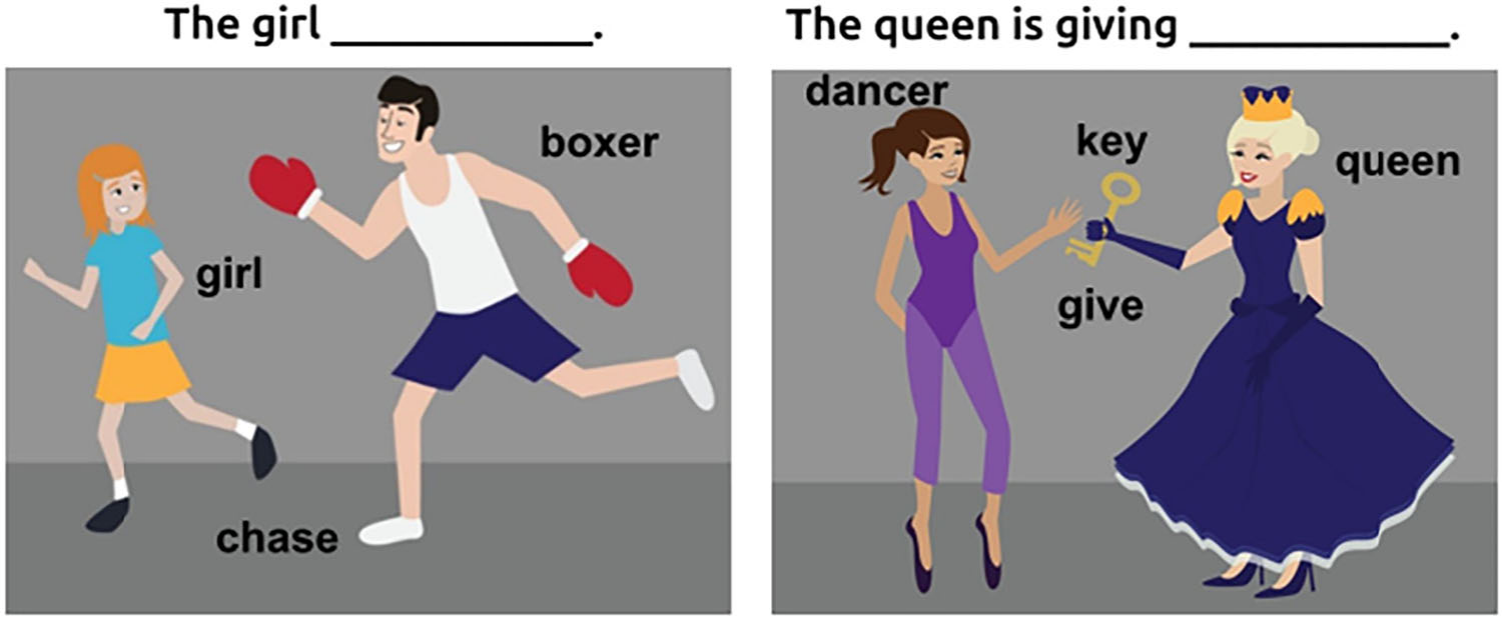
Example sentence production probe stimuli for passive (left) and dative (right) trials, administered at baseline and post–tests.

At baseline and post‐testing (sessions independent from training sessions), independent production of passives and DOs was assessed by administering a sentence production probe (30 passive and 30 dative items; see Figure 3). This task was divided into six blocks of ten trials each, with the order of blocks pseudo‐randomly presented across participants. Pictures were presented without a complete sentence frame (as in Figure 2). Instead, for passives only the first noun was presented above each picture (e.g., “The clown _________”) and for DO trials we presented the first noun and the main verb to be used (e.g., “The groom is giving______”). The verb was not presented in passive trials as this would have biased participants to produce a passive structure, while our current method allowed participants to generate passive or double‐object syntax independently. It was reasoned that if structural priming leads to improved access to passive and double‐object representations, participants should use more passives and double‐objects in their descriptions at post–testing than at baseline.

### 2.3 Procedure

After eligibility was confirmed, participants were baseline tested. Participants first completed the sentence production probes for passives and DOs. Then, they completed the sentence comprehension probe task. After eye movement calibration was conducted, they were told they would listen to sentences and select matching pictures. They were instructed to press ‘C’ to choose the left picture and ‘N’ for the right picture, and to respond as quickly as possible. Four practice trials (all comprising intransitive fillers) were presented first to ensure participants understood the task. Experimental trials were then presented in a fully randomized order. The task was divided into two blocks of 50 trials each, and participants were given the opportunity for a break following the first block. All tasks in all sessions were presented using Gorilla.sc (Anwyl‐Irvine et al. 2020).

#### 2.3.1 Training Task and Sentence Production Probes

**FIGURE 4:**
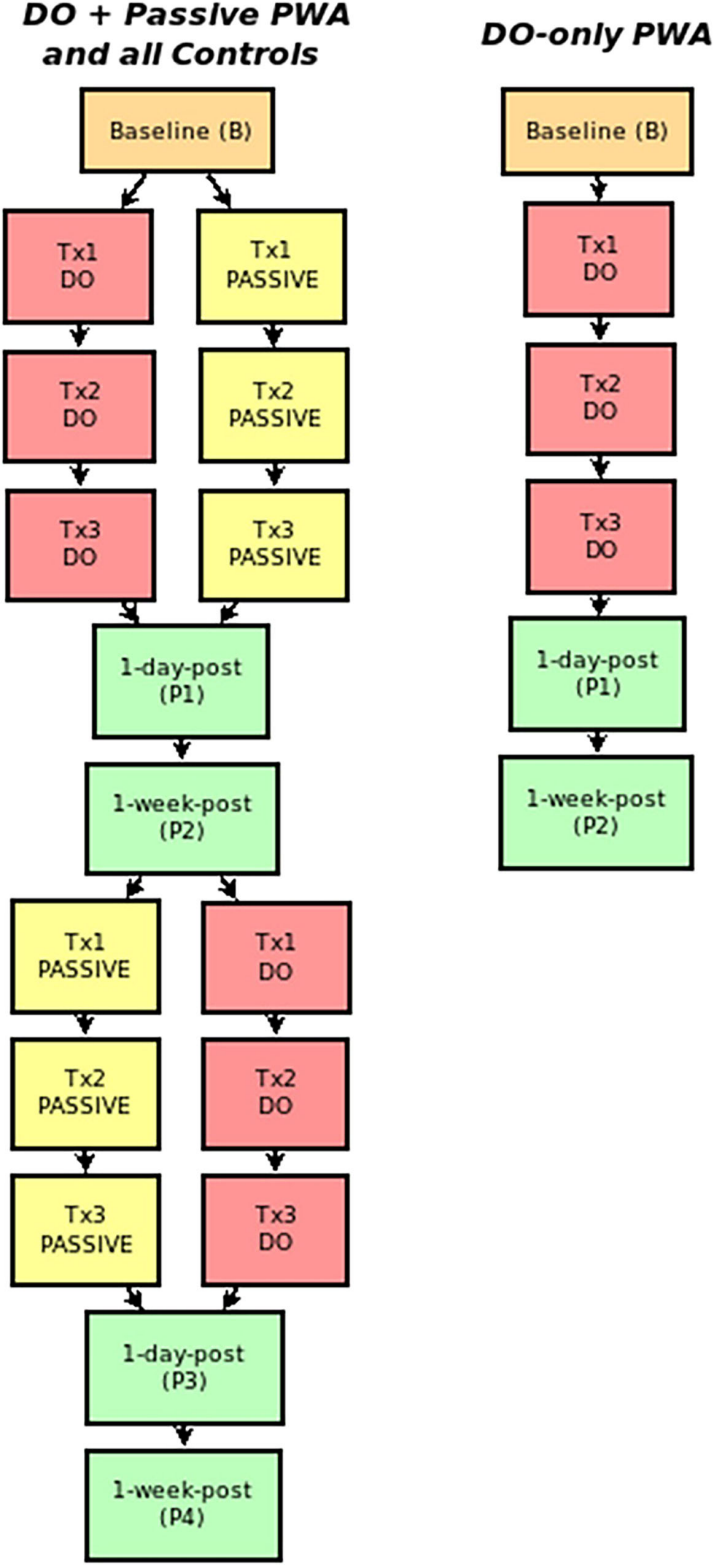
Schematic overview of training and post‐testing sequence.

Following baseline testing, participants were assigned to one of four training conditions, balancing the order of trained structures and same v. different verb training in a 2 × 2 design: (1) transitive‐different, dative‐same; (2) transitive‐same, dative‐different; (3) dative‐same, transitive‐different; (4) dative‐different, transitive‐same. Each structure was trained with three training sessions followed by an immediate (one day) post‐testing session and a delayed (one week) post‐testing session, which were identical to baseline sessions. Eight PWA scored at ceiling for passives on baseline sentence production measures, and were thus trained only on DOs (leaving 16 PWA to be trained on both structures). Four of these DO‐only PWA received same‐verb training and the other four received different‐verb training. See Figure 4 for a complete schematic overview of training sequences. Training sessions were spaced no more than four days apart, and training commenced within two weeks from baseline. No more than two weeks intervened between the first delayed post‐test (P2) and the fourth training session.

Training sessions consisted of a brief familiarization task in which participants repeated all nouns and verbs used in the training session to alleviate lexical retrieval difficulties in PWA. Then the priming training task followed. During priming training, participants were told that they would practice reading and making sentences. Prime sentences were presented in both writing and auditorily. Then, the participant was asked to read the prime sentence aloud. When they did not hear a recording (for target sentences), they were asked to complete the sentence using the words and sentence frame provided (Figure 2). Feedback was provided by experimenters only during reading of prime sentences. No feedback was given during target sentence completion. Sentence production probes at baseline and post‐testing were administered by presenting pictures one‐by‐one; participants made their sentences after experimenters named the words to use. To assess transfer of treatment gains to untrained items, in each probe task 40 trials that were trained during training sessions were presented (20 passive and 20 dative trials) along with 20 untrained exemplars (10 each of passive and dative).

#### 2.3.2 Eye Movement Recording (Comprehension Task)

During the comprehension task at baseline and post‐training testing, eye movements were recorded through participants’ own webcams using WebGazer.js (Papoutsaki et al., 2017). This algorithm, which has been integrated into the Gorilla.sc experimental software, takes participants’ webcam feeds and detects changes in the pixels corresponding to participants’ eyes. WebGazer outputs online predictions of participants’ eye gaze locations on the screen in X and Y co‐ordinates. Webcam‐based eye‐tracking has been validated for rough‐grained linguistic tasks in healthy participants as a reliable alternative to in‐person tracking (Slim and Hartsuiker 2023; Steffan et al. 2024), and our lab recently confirmed its applicability to aphasia research using a sentence‐picture matching task almost identical to that used in the present study (Van Boxtel et al. 2024; see also Van Boxtel et al. 2026).

Participants were calibrated twice prior to beginning the main comprehension task: first, before onset of practice trials, and again after the practice trials ended. Calibration procedures consisted of participants looking at colored dots on a five‐point grid, and each calibration was validated immediately afterwards. If validation determined that gazes for any calibration point were closer to a different point than the target point, calibration was repeated. Some PWA consistently failed to calibrate. Thus, a behavioral‐only task exactly mirroring the eye‐tracking task was administered wherever possible so behavioral data could still be recorded. Out of 24 PWA (104 sessions total), 8 sessions yielded behavioral data only (7.7%). Due to other recording issues, we were unable to record any data at all in six sessions for PWA, and three sessions for control participants.

### 2.4 Analysis

**FIGURE 5:**
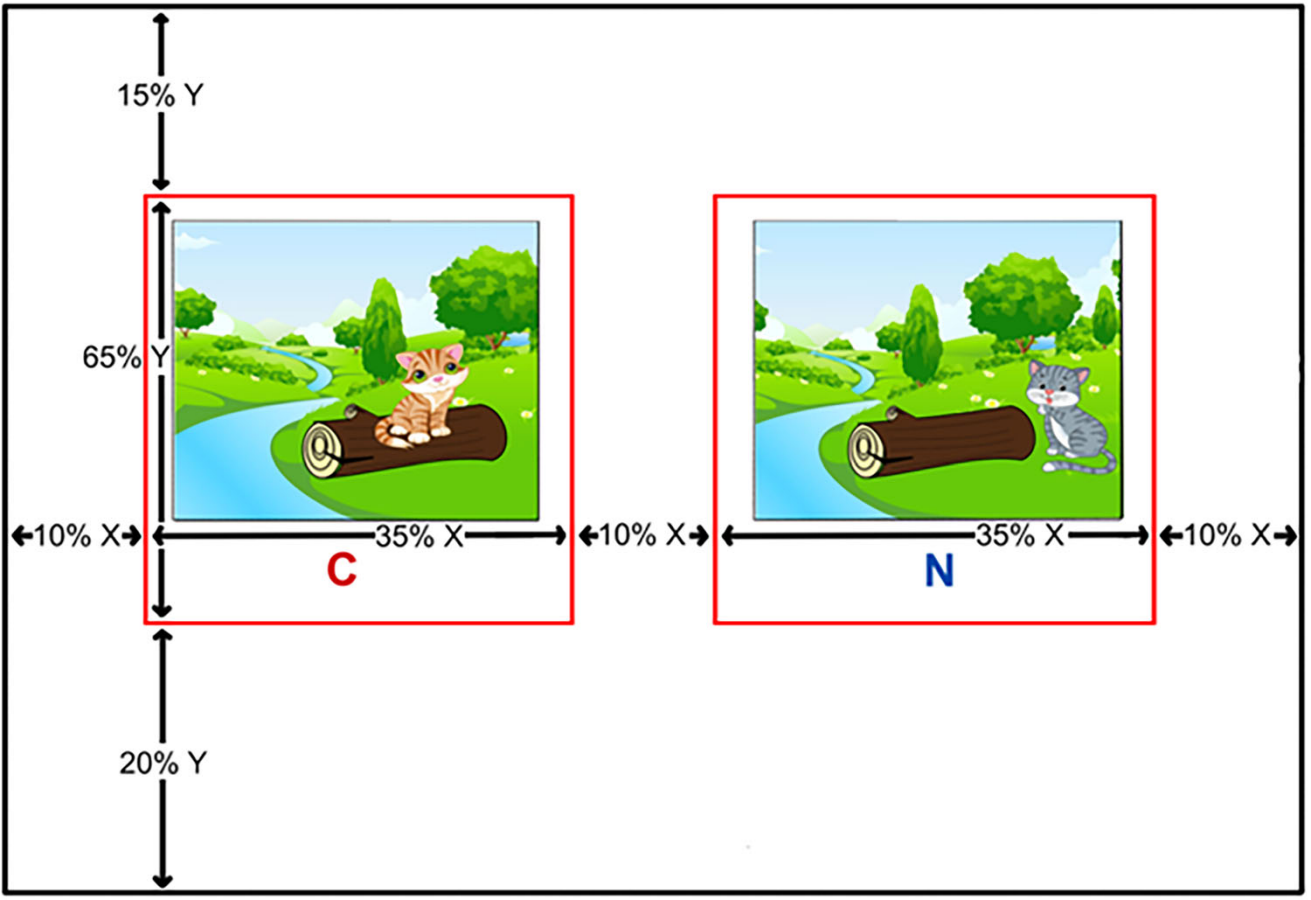
Proportionally computed AOI layout. For instance, for a 1920 × 1080px screen, the left AOI ranged from .10 * 1920 = 192px through to .45 * 1920 = 460.8px.

For the comprehension task, analyses centered on three main dependent variables: (a) accuracy, obtained as correct/incorrect responses in each trial; (b) response times (RT), measured as the interval between the end of the auditory sentence stimulus presented and a button press; (c) eye gazes as a binary target/foil factor, obtained during the entire trial duration. For eye gaze measures, target/foil Areas of Interest (AOIs) were defined proportionally to each participant's screen width in pixels. See Figure 5 for a visual overview of AOI computation. Both AOIs had a *y*‐axis range of 20% to 85% of pixels (to include gazes to ‘C’ and ‘N’ presented below each picture). The left picture AOI comprised between 10% and 45% of x‐axis pixels, while the right AOI was defined between 55% and 90% of x‐axis pixels. These AOIs allowed for variation between each participant's screen size (for instance, in our sample screen sizes ranged from 1200×800px to 2560 × 1440px) and the potentially reduced spatial accuracy of webcam‐based eye‐tracking compared to conventional eye‐tracking methods (Slim and Hartsuiker 2023).

Eye gaze samples were assigned either a target or foil label depending on which AOI they fell in (and which AOI, left or right, contained the target picture in any given trial). This binary measure served as our dependent variable for eye gaze data. All incorrect trials and observations that fell outside either the target or foil AOI were removed from the eye data analysis.

To evaluate whether sentence production improvements were associated with improvements in comprehension, we also assessed accuracy on baseline and post‐testing sentence production probes. Verbal responses were transcribed verbatim by trained coders and scored for accuracy: passive targets were scored correct if theme, verb, and agent appeared in the correct position and passive morphology (‘by’ + ed) was produced. DO targets were scored correct if agent, verb, theme, and goal were produced in the correct position, while verb tense was allowed to vary (e.g., “The groom [is/was giving]/[gave] the artist the coffee”). If multiple attempts were made, the final sentential attempt was scored. Word substitutions that did not alter the meaning of the target sentence (e.g., chef/cook, man/guy) were accepted. Further, for PWA the omission of auxiliary verbs and articles, or intelligible sound errors were accepted (see Lee et al. 2025 for detailed accuracy and error coding procedures). Inter‐coder reliability of 94.5% agreement was achieved on randomly selected 15% of the data. To relate production accuracy improvements to comprehension performance, we computed a production accuracy gain measure by subtracting mean production accuracy at each participant's last post–testing session from mean production accuracy at baseline: ∆Prod = M(P2 or P4 Accuracy) − M(Baseline Accuracy)

#### 2.4.1 Statistical Methods

All statistical analyses were run in R v.4.3.1 (R Core Team 2023) and RStudio v.2023.06.0 (RStudio Team 2020). We used the *tidyverse* (Wickham et al. 2019), *data*.*table* (Dowle et al. 2019), stringr (Wickham 2022), and *readxl* (Wickham et al. 2023) packages for general data preprocessing, *ggplot2* (Wickham 2016) for data visualization, and *fitdistrplus* (Delignette‐Mueller and Dutang 2015) to evaluate data normality. To build linear and binomial mixed‐effects regression models, we used the *lme4* (Bates et al. 2015) and *lmerTest* (Kuznetsova et al. 2017) libraries, and generated additional model statistics using *MuMIn* (Barton and Barton 2015), *EMAtools* (Kleiman 2021), and *emmeans* (Lenth 2017). Finally, the *mgcv* (Wood 2011) and *itsadug* (Van Rij et al. 2022) packages were used for generalized additive mixed modelling. Accuracy was assessed using binomial mixed‐effects models, while RT was modeled with a linear distribution. The RT data were trimmed to improve normal fit by excluding datapoints more than two standard deviations away from each participant's mean. Remaining RTs were log‐transformed. Our eye gaze measure was examined using generalized additive mixed‐effects models (GAMMs), which have become a standard tool for accurately modeling time series data, especially when data consist of non‐linear patterns (Wieling 2018; Wood 2017). GAMMs further have the added benefit of controlling for autocorrelation between time points. For our eye gaze measure, binomial GAMMs were used to reflect the binary factorial outcome variable. Each model included a three–way interaction term between group (PWA v. control), session (baseline v. immediate post–testing [P1] v delayed post–testing [P2]), and training condition. Both RT and eye data analyses excluded incorrect trials.

Wherever possible, models were fit with a maximal random effects structure consisting of random slopes of trial by participant. If this structure failed to converge, separate random intercepts of trial and participant were fit, and if either of these terms resulted in convergence failures, the intercept for trial was removed before the intercept for participant. In our GAMM analyses, all fixed and random effects were fitted in a non–linear fashion (called “smooths”), and if GAMMs did not show accurate fit, the number of basic functions (called knots, or *k*) was increased.

## 3 Results

We first report effects on sentence comprehension accuracy in section 3.1, and on response times in section 3.2. We report an investigation into whether gains in sentence production predicted gains in sentence comprehension in section 3.3, followed by our analysis of eye gaze data in section 3.4.

### 3.1 Sentence Comprehension Accuracy

**FIGURE 6:**
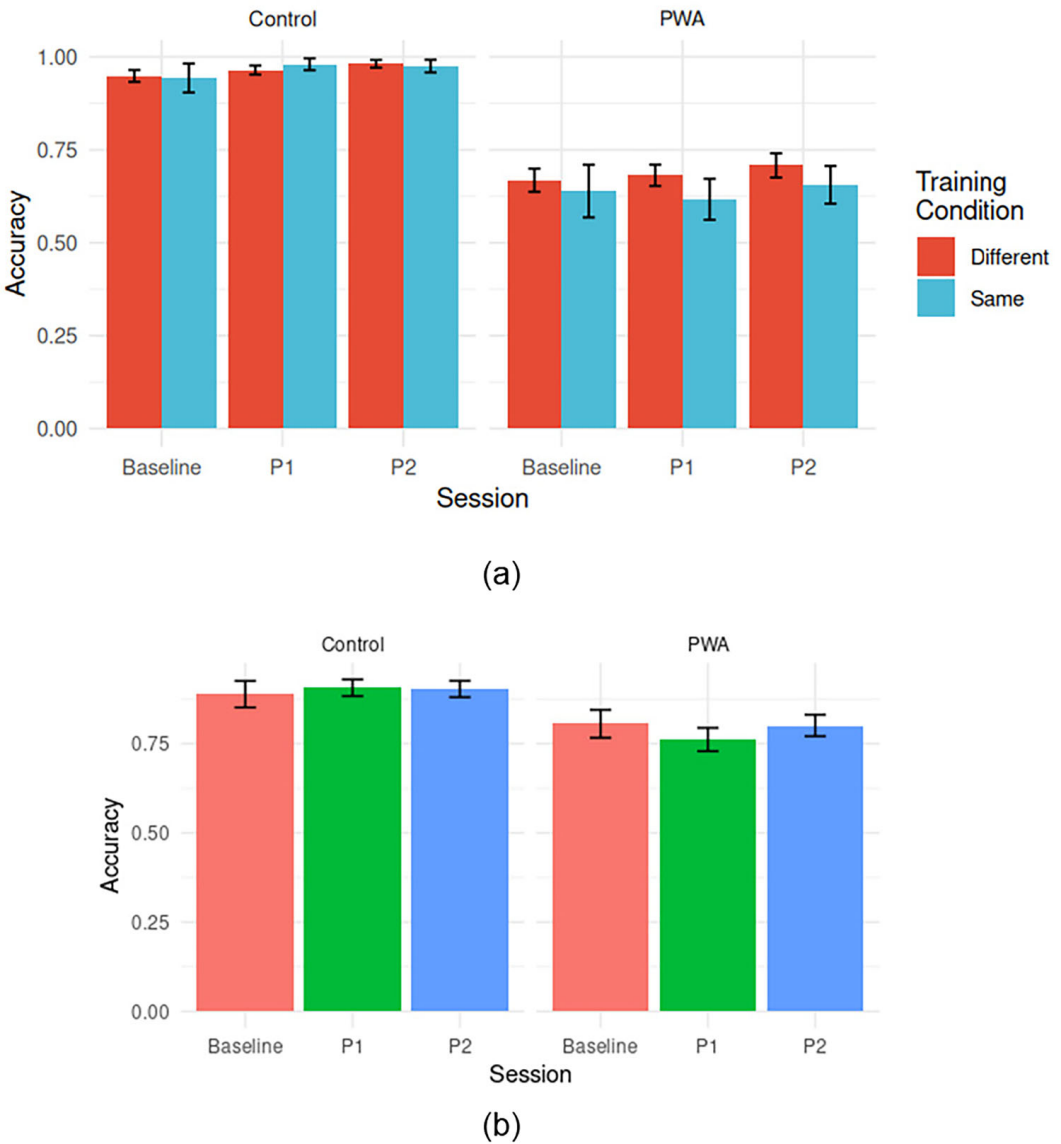
Accuracy by session and training condition, critical trials only (a); and accuracy by session on control (locative) trials (b).

Plots illustrating accuracy by session and training condition in critical (passive and DO dative) and filler trials are given in Figure 6a andb, respectively. A binomial mixed‐effects model was fitted to critical trials with random effects of group, session, and training condition (including a random effect of participant; other random effects led to model convergence problems). Session was contrast coded to compare P1 v. baseline, and P2 v. baseline. Accuracy did not increase between sessions (*p*s > .05), and there were no effects of training condition (all *p*s > .05). See Table 2, upper panel, for full model output. We subsequently modeled PWA data separately (Table 2, middle panel), which demonstrated no overall improvement between sessions (*p*s > .05), but overall lower accuracy in the same compared to the different condition (*z* = ‐4.468, *p* < .001, OR = .547). Finally, we modeled accuracy on control (locative) structures (see Figure 6b and lower panel of Table 2). Accuracy on locatives did not change between sessions (*p*s > .05), but PWA were less accurate than controls overall (*z* = ‐5.200, *p* < .001).

**TABLE 2:** Binomial mixed model output of accuracy models (Accuracy ∼ Group * Session * Condition). Model for All data (R^2^_Marginal_ = .306, R^2^_Conditional_ = .492) was fitted with a random intercept for Participant (σ^2^ = 1.196, SD = 1.094). Model for PWA only (R^2^_Marginal_ = .012, R^2^_Conditional_ = .356) included random intercepts for Participant (σ^2^ = 1.426, SD = 1.194) and Trial (σ^2^ = .224, SD = .474). Model for Locatives (R^2^_Marginal_ = .080, R^2^_Conditional_ = .513) included random intercepts for Participant (σ^2^ = .442, SD = .665) and Trial (σ^2^ = 2.480, SD = 1.575). Abbreviations: SE = standard error; OR = odds ratio; Lower/Upper CI: 97.5% confidence intervals.

Data	Parameter	β	SE	*Z*	*P*	OR	Lower CI	Upper CI
All data	Intercept	3.864	.343				4.356	4.536
	Group: PWA	−2.813	.420	−6.703	**< .001**	.060	−3.635	−1.990
	B v. P1	‐.146	.215	‐.680	.496	.864	‐.568	.275
	B v. P2	.416	.347	1.200	.230	1.515	‐.264	1.095
	Training: Same	.085	.300	.285	.776	1.089	‐.502	.672
	PWA * B v. P1	.204	.225	.909	.363	1.227	‐.237	.645
	PWA * B v. P2	‐.539	.356	−1.513	.130	.058	−1.237	.159
	PWA * Same	‐.329	.324	−1.018	.309	.079	‐.963	.305
	B v. P1 * Same	.312	.341	.917	.359	1.367	‐.355	.980
	B v. P2 * Same	‐.333	.430	‐.775	.438	.717	−1.174	.509
	PWA * B v. P1 * Same	‐.334	.365	‐.916	.360	.716	−1.049	.381
	PWA * B v. P2 * Same	.588	.452	1.301	.193	1.800	‐.298	1.474
PWA only	Intercept	1.194	.281				.643	1.746
	B v. P1	.027	.068	.392	.695	1.027	‐.107	.161
	B v. P2	‐.021	.088	‐.236	.814	.980	‐.192	.151
	Training: Same	‐.603	.135	−4.468	**< .001**	.547	‐.868	‐.339
	B v. P1 * Same	.042	.132	.321	.748	1.043	‐.216	.300
	B v. P2 * Same	.170	.142	1.196	.232	1.857	‐.109	.449
Locatives	Intercept	3.230	.412				2.422	4.037
	Group: PWA	−1.385	.266	−5.200	**< .001**	.250	−1.908	‐.863
	B v. P1	.123	.160	.767	.443	1.130	‐.191	.436
	B v. P2	.077	.156	.49	.621	1.080	‐.228	.382
	PWA * B v. P1	‐.328	.190	−1.725	.085	.721	‐.700	.045
	PWA * B v. P2	.022	.188	.116	.906	1.022	‐.347	.391

We briefly explored whether substantial individual variability was present in our accuracy data, which may have caused a ‘washing out’ of the main effects. There was substantial variability in the PWA group especially. We have included a table summarizing individual comprehension accuracy in all PWA participants in our supplementary materials at https://osf.io/rhbue/. Overall, 14 PWA showed comprehension accuracy improvement from baseline to post‐testing (either at P1 or P2), while 10 showed little to no improvement. This variability likely explains why no overall accuracy gains were significant in the by‐group analysis.

### 3.2 Response Times

**FIGURE 7:**
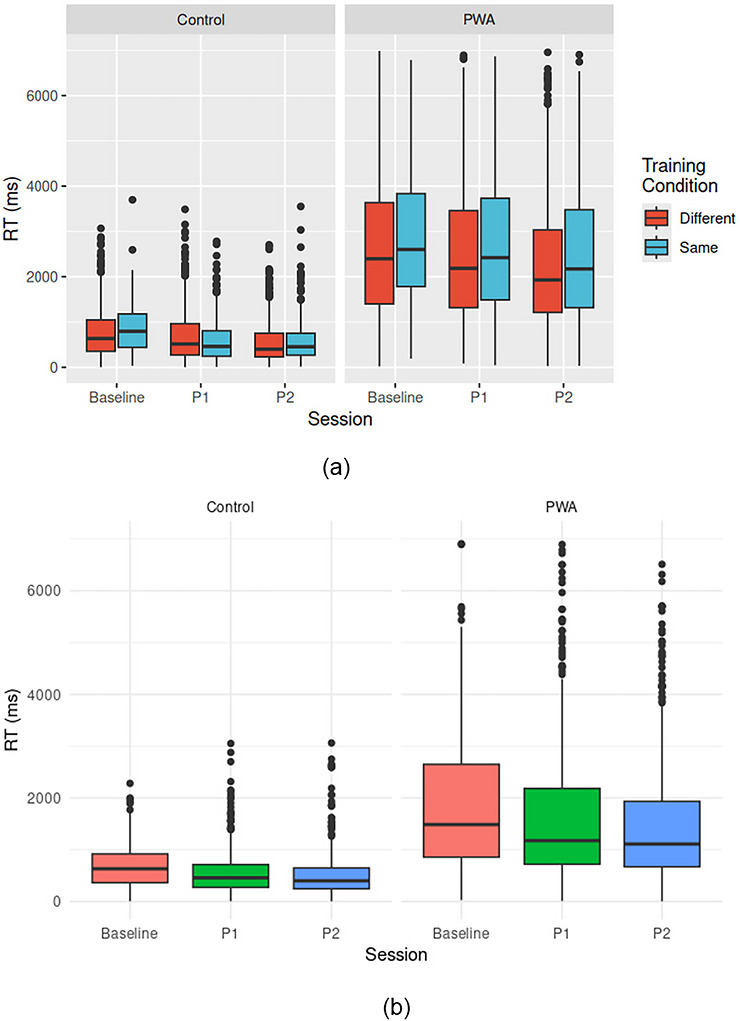
Plots showing response times to critical trials (a) and control (locative) trials (b).

**FIGURE 8:**
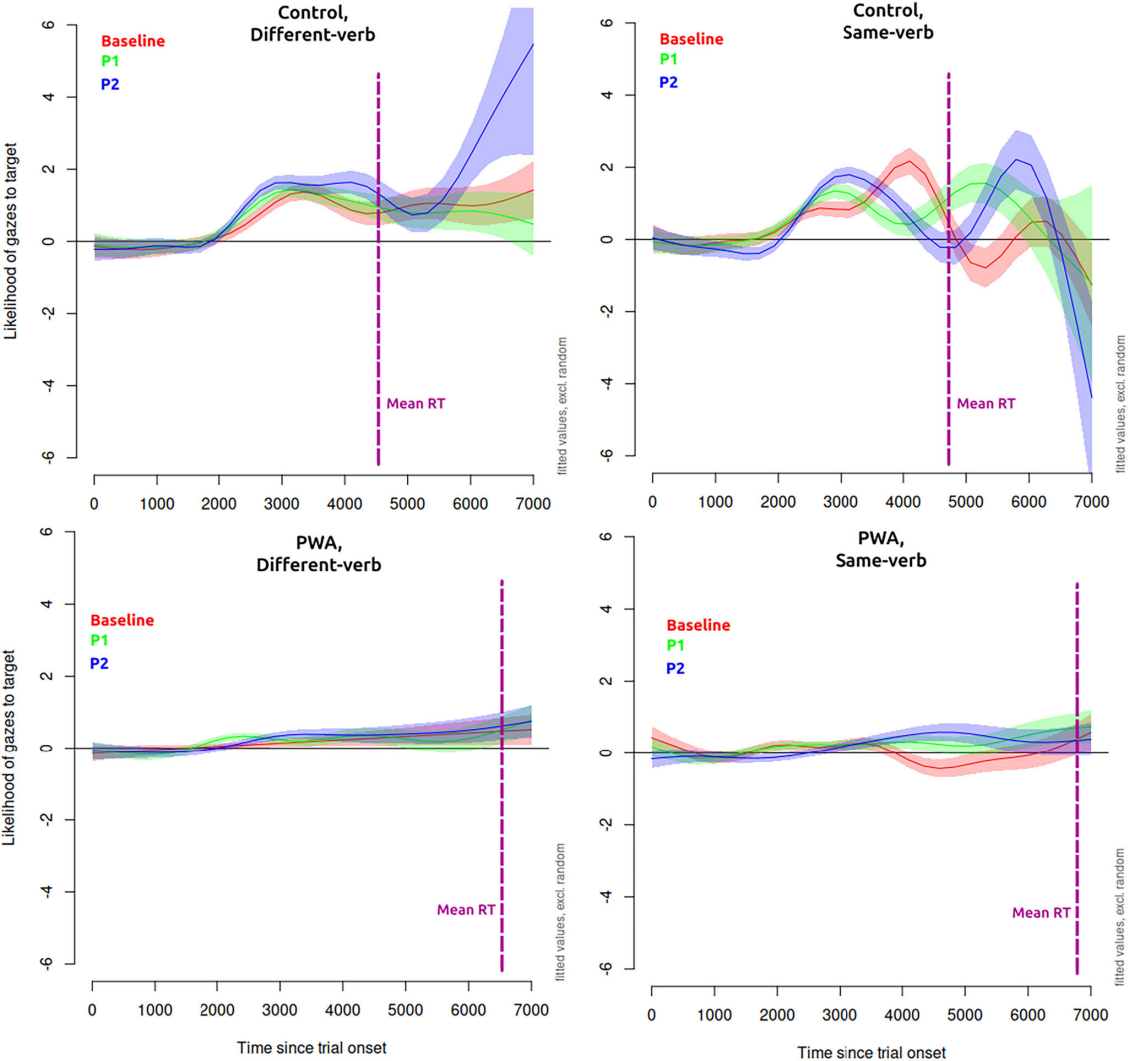
Non–linear smooth visualizations of gazes to the target picture by group, session, and training condition, showing larger differences for controls in the same–verb compared to the different–verb condition, but *smaller* differences for PWA in the same–verb compared to the different–verb condition.

Response times (RTs) for picture matching were also assessed for improvements between sessions, by group and training condition. For full model output, refer to Table 3. Visualizations of RTs in critical trials are given in Figure 7a, while effects on our control (locative) trials are illustrated in Figure 7b. On critical trials, PWA took longer to respond than controls overall (*t* = 9.467, *p* < .001, OR = 4.376), and while there was overall improvement between baseline and delayed post‐testing (*t* = ‐9.326, *p* < .001, OR = .800), this improvement was less strong in PWA compared to controls (*t* = 3.724, *p* < .001, OR = 1.142). RTs reduced marginally overall from baseline to immediate post‐testing (*p* = .08). Crucially, this overall improvement from B to P1 was affected by training condition: RTs were facilitated by the same–verb condition more so than the different–verb condition (*t* = ‐3.861, *p* < .001, OR = .837). However, this effect was reversed in PWA (see Figure 8a), who showed shorter RTs in the different–verb condition (*t* = 2.096, *p* < .05, OR = 1.161). This suggests controls benefited more from same–verb training, while PWA showed greater improvements following different–verb training. Response times to locatives similarly decreased between baseline and delayed post–testing (*t* = ‐6.220, *p* < .001, OR = .820). On control (locative) trials, PWA were slower than controls overall (*t* = 7.914, *p* < .001, OR = 2.741), but both groups showed similar by‐session response time changes (*p*s > .05). To ensure the locative stimuli could still be considered an accurate control for our priming effects, we additionally built a model assessing training condition effects on RTs for locative trials. This model is reported in detail in our supplementary materials (https://osf.io/rhbue/), and included a two‐level factor for locative v. critical trials. Briefly, critical (passive and DO wh‐questions) trials showed a greater effect of same v. different‐verb training conditions, compared to locative control trials (2‐ way interaction *t* = 2.322, *p* < .05, OR = 1.104). This interaction suggests that the observed changes in our passive and DO wh‐questions are unlikely due to repeated exposure of the wh‐questions. The supplementary materials further include figures and a model summary detailing these effects.

**TABLE 3:** Linear mixed model output of reaction time (RT) models (LogRT ∼ Group * Session * Condition). Abbreviations: SE = standard error; OR = odds ratio; Lower/Upper CI: 97.5% confidence intervals. Models included random intercepts for Participant (critical trials: σ^2^ = .219, SD = .468; locatives: σ^2^ = .139, SD = .373) and Trial (critical trials: σ^2^ = .034, SD = .183; locatives: σ^2^ = .057, SD = .239). R^2^_Critical, Marginal_ = .410; R^2^_Critical, Conditional_ = .595; R^2^_Locatives, Marginal_ = .248; R^2^_Locatives, Conditional_ = .416.

Data	Parameter	β	SE	*t*	*P*	OR	Lower CI	Upper CI
Critical trials	Intercept	6.146	.122				5.908	6.385
	Group: PWA	1.476	.156	9.467	**< .001**	4.376	1.171	1.782
	B v. P1	.038	.022	1.726	.084	1.039	‐.005	.082
	B v. P2	‐.223	.024	−9.326	**< .001**	.800	‐.269	‐.176
	Training: Same	.055	.035	1.551	.121	1.056	‐.014	.124
	PWA * B v. P1	‐.011	.033	‐.322	.747	.989	‐.076	.055
	PWA * B v. P2	.133	.036	3.724	**< .001**	1.142	.063	.202
	PWA * Same	‐.015	.055	‐.272	.786	.985	‐.123	.093
	B v. P1 * Same	‐.178	.046	−3.861	**< .001**	.837	‐.268	‐.087
	B v. P2 * Same	.067	.046	1.440	.150	1.069	‐.024	.157
	PWA * B v. P1 * Same	.149	.071	2.096	**.036**	1.161	.010	.289
	PWA * B v. P2 * Same	‐.107	.071	−1.508	.132	.899	‐.245	.032
Locatives	Intercept	6.139	.111				5.922	6.356
	Group: PWA	1.008	.124	7.914	**< .001**	2.741	.759	1.258
	B v. P1	‐.039	.032	−1.204	.229	.962	‐.102	.024
	B v. P2	‐.198	.032	−6.220	**< .001**	.820	‐.260	‐.136
	PWA * B v. P1	.030	.046	.659	.510	1.031	‐.060	.120
	PWA * B v. P2	.059	.045	1.245	.213	1.057	‐.032	.144

### 3.3 Relating Production Improvements to Comprehension

Sentence production probes were taken at baseline and both post–tests prior to administration of the sentence comprehension task (see Section 2.4 for details), and we aimed to test if priming–induced improvements on sentence production significantly associated with changes in the response times (RT) in the comprehension task. We first confirmed the efficacy of our training in production, that is, whether sentence production accuracy increased following training, building a generalized mixed–effects model with production accuracy as dependent variable. This model is summarized in our supplementary materials (Table 1; https://osf.io/rhbue/) and detailed production results are reported in Van Boxtel et al. (2025). Briefly, participants’ sentence production accuracy increased overall from baseline (M_Control_ = .537; M_PWA_ = .351) to P1 (M_Control_ = .740; M_PWA_ = .493; *z* = 4.826, *p* < .001, OR = 1.418) and to P2 (M_Control_ = .818; M_PWA_ = .523; *z* = 11.197, *p* < .001, OR = 2.458). While improvement from B to P2 was less strong in PWA than in controls (*z* = ‐4.573, *p* < .001, OR = .629), no effects of exposure of trials during training were found anywhere (all *p*s > .05), indicating full transfer from trained to untrained exemplars.

To relate improvements in production to those in comprehension, we computed a production difference score (∆Prod) by subtracting each participant's mean accuracy at baseline from their mean accuracy at their last post–test (see Section 2.5). This ∆Prod value was then used as a predictor in comprehension accuracy and RT (response time) models. Models including a three–way session * group * ∆Prod interaction fit the data better than null models containing only a main effect of ∆Prod for both accuracy data (*χ* ^2^ (5) = 11.55, *p* = .04) and response time data (*χ* ^2^ (5) = 27.72, *p* < .001). The accuracy model, however, showed no significant interaction terms of session or group by ∆Prod, and as such this model is reported only in our supplementary materials (Table 2; https://osf.io/rhbue/). The RT model is summarized here in Table 4. Across groups, participants who improved more on production probes (higher ∆Prod scores) also showed greater reaction time gains between baseline and P1 (*t* = ‐2.994, *p* < .01, OR = .625) and between baseline and P2 (*t* = ‐2.466, *p* < .05, OR = .668). This effect was less strong in PWA from baseline to P2 (*t* = 2.531, *p* < .05, OR = 1.990).

**TABLE 4:** Linear mixed model output of Δ_Prod_ reaction time (RT) models (Δ_Prod_ ∼ Session * Group). Abbreviations: SE = standard error; OR = odds ratio; Lower/Upper CI: 97.5% confidence intervals. Model included random intercepts for Participant (σ^2^ = .223, SD = .475) and Trial (σ^2^ = .034, SD = .183). R^2^_Marginal_ = .412; R^2^_Conditional_ = .600.

Parameter	β	SE	*Z*	*P*	OR	Lower CI	Upper CI
Intercept	6.23	.184				5.870	6.592
B v. P1	.063	.030	2.127	**.033**	1.065	.005	.122
B v. P2	‐.138	.031	−4.503	**< .001**	.871	‐.198	‐.078
Group: PWA	4.456	.233	6.259	**< .001**	4.290	1.000	1.912
Δ_Prod_	‐.503	.976	‐.516	.609	.604	−2.415	1.409
PWA * B v. P1	‐.030	.042	‐.707	.480	.971	‐.111	.052
PWA * B v. P2	.012	.044	.267	.789	1.012	‐.075	.098
B v. P1 * Δ_Prod_	‐.470	.157	−2.994	**.003**	.625	‐.778	‐.162
B v. P2 * Δ_Prod_	‐.403	.164	−2.466	**.014**	.668	‐.724	‐.083
PWA * Δ_Prod_	‐.035	1.351	‐.026	.979	.966	−2.682	2.613
PWA * B v. P1 * Δ_Prod_	.354	.257	1.378	.168	1.425	‐.150	.858
PWA * B v. P2 * Δ_Prod_	.688	.272	2.531	**.011**	1.990	.155	1.221

### 3.4 Eye Movements

These models evaluated whether group (PWA and controls), session (Baseline, P1, and P2), and training condition (same‐ and different–verb) affected the likelihood of gazes to either target or foil. For this, the target/foil binary factor was modeled numerically (with 1 representing target and 0 representing foil gazes) as a function of time since trial onset, group, session, and training condition. A model including the three–way interaction of group * session * training condition yielded better model fit than a model including a two–way interaction of group * session only (AIC difference = 758.65). A non–linear random intercept for participant was included in the final model. See Table 5 for summaries of parametric effects and smooth terms from the final model. Eye movement models included critical trials only (excluding locative trials), as behavioral data suggested little to no training condition effects on locative items.

**TABLE 5:** Generalized additive mixed model output of binary target/gaze factor model (Gaze Location ∼ Group * Session * Condition). Abbreviations: SE = standard error; EDF = effective degrees of freedom; Ref.df = reference degrees of freedom (for computing test statistics).

Parametric coefficients	β	SE	*t*	*p*
Intercept	.393	.049		
PWA * Baseline * Different	‐.323	.065	−4.997	**< .001**
Control * P1 * Different	.087	.016	5.575	**< .001**
PWA * P1 * Different	‐.279	.065	−4.329	**< .001**
Control * P2 * Different	.260	.025	10.360	**< .001**
PWA * P2 * Different	‐.281	.065	−4.318	**< .001**
Control * Baseline * Same	.019	.027	.692	.489
PWA * Baseline * Same	‐.322	.067	−4.784	**< .001**
Control * P1 * Same	.048	.027	1.786	.074
PWA * P1 * Same	‐.266	.066	−4.052	**< .001**
Control * P2 * Same	.013	.025	.529	.597
PWA * P2 * Same	‐.342	.066	−5.218	**< .001**
				
Smooth terms	EDF	Ref.df	*χ* ^2^	*p*
s(Time,Control*Baseline*Different)	8.313	8.626	112.861	**<001**
s(Time, PWA*Baseline*Different)	1.063	1.080	4.731	**.034**
s(Time, Control*P1*Different)	7.852	8.336	123.971	**<001**
s(Time, PWA*P1*Different)	8.038	8.701	104.323	**<001**
s(Time, Control*P2*Different)	8.188	8.600	207.596	**<001**
s(Time, PWA*P2*Different)	6.049	7.175	45.103	**<001**
s(Time, Control*Baseline*Same)	8.810	8.962	165.798	**<001**
s(Time, PWA*Baseline*Same)	8.007	8.707	95.420	**<001**
s(Time, Control*P1*Same)	7.946	8.457	136.358	**<001**
s(Time, PWA*P1*Same)	7.277	8.233	40.846	**<001**
s(Time, Control*P2*Same)	8.703	8.929	331.311	**<001**
s(Time, PWA*P2*Same)	6.199	7.322	64.537	**<001**
s(Time, Participant)	287.484	331	7221.774	**<001**

PWA showed lower likelihoods of gazing at the target than controls overall (βs < ‐.25, ts < ‐4, *p*s < .001). For the interpretation of other effects, though, evaluation of non–linear smooths was necessary. For these non–linear smooths, EDF represents how non–linear each smooth term is (with EDF = 1 representing a fully linear pattern and higher values suggesting greater degrees of non–linearity). The significance tests for non–linear terms suggest there are reliable differences between groups, sessions, and training conditions as a function of time, and that modeling non—linear smooths was necessary to most accurately capture the data. See Baayen and Linke (2021) and Baayen et al. (2017) for overviews of the generalized additive mixed model for human subjects research, and Wieling (n.d.) for a practical tutorial.

For a more detailed interpretation of additive models and to effectively consider non–linear terms, visualization is critical. In Figure 8, non–linear smooths by group, session, and training condition are presented. Average likelihoods of gazes to the target picture increased as trials progressed, indicating that both groups focused on the target picture after disambiguation. Generally, PWA showed weaker effects compared to controls (compare the two upper panels and the two lower panels in Figure 8), which also aligns with our response time findings. However, PWA showed greater by–session differences in the different–verb compared to the same–verb condition, with greater likelihoods of gazes to the target at P1 and P2 compared to baseline in the different–verb condition from around 3000 ms post‐onset. Controls, on the other hand, show greater changes in the same‐verb condition compared to the different‐verb condition (compare the upper left and upper right panels of Figure 8).

## 4 Discussion

This study aimed to capture cross–modality generalization from sentence production to sentence comprehension following structural priming treatment in persons with aphasia (PWA). Additionally, we asked whether generalization is affected by lexical overlap between primes and targets during sentence production training, and whether gains in production directly predict the magnitude of generalization to comprehension.

### 4.1 Cross–Modality Generalization

Our first research question asked whether structural priming training in production yielded cross‐modality generalization to sentence comprehension. We hypothesized, following past research on structural priming across modalities, that priming training in production should lead to accuracy and response time facilitation on our comprehension task from baseline to post‐testing. Additionally, we expected that participants should show faster and greater eye fixations to the target picture at post‐testing compared to baseline.

The structural priming training task resulted in reliable treatment gains in sentence production. Crucially, some treatment gains were also found on certain comprehension measures, suggesting some cross–modality generalization did occur. While no reliable changes to comprehension accuracy were detected, response times to comprehension trials decreased by roughly 300 ms on average for PWA between baseline and immediate post–testing, and both PWA and controls fixated the correct (target) picture more (and more quickly) at post–testing compared to baseline. We therefore still suggest structural priming may be an effective tool for achieving cross–modality treatment generalization from production to comprehension in aphasia, especially in online indices of comprehension.

The present findings may concur with accounts of close alignment between the production and comprehension modalities, at least at the representational levels tapped by structural priming (e.g., Dickey and Yoo 2010; Segaert et al. 2012; Matchin et al. 2023). Our findings further suggest structural priming could be developed into an effective treatment for sentence production and sentence comprehension deficits in aphasia, in line with previous explorations of this method in PWA (Lee and Man 2017; Lee 2024; Lee et al. 2025; Keen and Lee 2024; Rainey et al. 2026; Saffran and Martin 1997). Past literature already established that structural priming occurs across modalities in single‐session studies (e.g., Bock et al. 2007; Keen and Lee 2024 for PWA). However, this study is among the first to demonstrate some cross‐modality training gains in PWA following structural priming training, and to directly associate improvements in sentence production with improvements in sentence comprehension. Our second research question centered on this aim, and asked whether improvements in sentence production are tied directly to improvements in sentence comprehension. This association was tested using our ∆Prod measure. Our results suggest that participants who showed greater improvement in production accuracy following training also showed greater reduction in response times in comprehension measures, evidencing cross–modality generalization. Identifying parameters that can amplify such cross‐modality generalization gains will be important for future research on approaches to aphasia rehabilitation. Further, applying structural priming to formal treatment protocols, and determining the specific learning mechanisms underlying priming, will be two necessary future steps to achieving treatment gains in production and cross–modality generalization.

Prior studies of cross‐modality treatment generalization in PWA have found limited and inconsistent effects (see Adelt et al. 2018, for a review), and indeed, our accuracy findings add to this list. Further, those studies that do report reliable generalization mostly found effects from comprehension to production, with production–to–comprehension generalization notably absent from existing literature (Jacobs and Thompson 2000; Poirier et al. 2023). We nevertheless suggest we found evidence of generalization from production to comprehension using our response time and eye movement measures. We highlight several potential differences between past studies and this experiment to help explain our findings: first, the present study differed from most past investigations in its use of structural priming, an implicit technique targeting syntactic representations while minimizing confounds of impaired short‐term memory, attentional deficits, or declarative skills (Chang et al. 2012; Heyselaar et al. 2021). Despite null effects of verb overlap in PWA (see section 4.2), structural priming did improve both production and some comprehension metrics in this study, suggesting that priming could be a more robust and generalizable treatment component than existing programs. In addition, past studies have demonstrated reliable cross‐modality transfer of structural priming effects from production to comprehension and vice versa in healthy adults (e.g., Litcofsky and van Hell 2019; Branigan et al., 2005). This supports the notion that the learning observed in priming reflects changes to structural representations beyond or above the level of individual modalities.

Second, while most past studies of generalization focus mainly on behavioral response accuracy (either in production or comprehension), the present study found its generalization effects in more fine‐grained measures, response times and eye movement data. We failed to find significant improvement in comprehension accuracy, likely in part due to substantial individual variability. The current training paradigm, where participants received a relatively small number of training sessions in production, may thus not be sufficient to improve offline comprehension data. Accuracy measures for sentence production and comprehension necessarily capture less of the processes underlying encoding and processing than more fine–grained metrics and sub–behavioral measures (Godfroid et al. 2020; Mulder and van Maanen 2013; Prinzmetal et al. 2005; Sharma et al. 2021). Accuracy metrics in the present study showed little to no overall change between sessions, even though response times and eye movements did reveal generalization patterns. Possibly, this is due to the complexity of, and difficulty associated with, the stimuli used in our experiment. Passive structures are notoriously difficult for PWA to comprehend successfully (see, among many others, Berndt et al. 1997; Friederici and Graetz 1987; Man et al. 2019; Meyer et al. 2012). Similarly, past studies have shown significant impairments in the processing and encoding of double–object datives in PWA (e.g., Caplan and Hanna 1998; Man et al. 2019). Given these findings, it is perhaps less surprising that this study did not find accuracy improvements—nevertheless, the robust results we observed in response times and eye movements evidence substantial treatment effects. It will be worthwhile for future research to examine how generalization to offline measures such as accuracy can be strengthened. For instance, the current study only included up to six treatment sessions; if more sessions of structural priming training are included in future programs, cross‐modality generalization may become more apparent on offline accuracy measures. Relatedly, future research should address the substantial inter‐participant variability we uncovered in our offline accuracy measures: it will be of critical importance to determine why some PWA showed greater generalization than others.

Finally, this study captured eye movements effectively using webcam–based eye–tracking. This mirrors previous results from healthy populations (e.g., Slim and Hartsuiker 2023) and tentative work from our lab in PWA (Van Boxtel et al. 2024), and makes the case for more widespread use of webcam–based eye–tracking in clinical groups. Participants in this study resided in an area with a radius of around 2000 km (1500 miles), spanning four time zones, but still participated and provided accurate and sensitive sub–behavioral data, which contributed greatly to the robustness of our cross–modality generalization findings. All participants could wear glasses without interfering with corneal reflections or pupil capture (which are, indeed, not required for WebGazer's tracking method), were recorded without the use of expensive equipment, and could conduct the experiment from the comfort of their own homes. This represents a crucial advance in the recording and analysis of eye movements, especially in clinical populations such as PWA, who often exhibit reduced mobility and a wide geographic distribution, and we suggest future research should continue to use and validate online eye–tracking with as many different samples and clinical groups as possible.

### 4.2 Manipulating Verb Overlap

Our third and final research question concerned the widely evidenced facilitation of structural priming by verb overlap between prime and target sentences, known as the lexical boost (e.g., Hartsuiker et al. 2008). We hypothesized that same‐verb structural priming training would lead to stronger cross‐modality generalization than different‐verb priming training. To test this hypothesis and evaluate whether lexical boost effects facilitate priming–induced acquisition and generalization to comprehension, participants were trained with same–verb and different–verb priming blocks. Control participants exhibited the hypothesized lexical boost effects: the same–verb condition resulted in faster response times at post–testing compared to the different–verb condition, and changes in gazes to the target picture were greater between sessions in the same–verb condition. These findings concur with past results of intact lexical boost effects in healthy older adult groups (e.g. Tooley 2020; Van Boxtel and Lawyer 2023a,b).

Conversely, PWA were more facilitated by different–verb training on both response times and eye movement measures, in a notable contrast to healthy controls. Previous studies have found inconsistent effects of lexical overlap on priming effects in PWA: while some studies found amplified priming with lexical overlap in aphasia (e.g., Lee et al., 2023; Yan et al. 2018), this study aligns with reports which support an absence of the lexical boost in PWA (e.g., Lee et al. 2019a; Man et al. 2019; Van Boxtel et al. 2023a). This absence suggests that different learning conditions facilitate priming in PWA compared to neurologically healthy controls. While controls used lexical information to amplify priming, PWA did not use lexical strategies. This could be a result of frequently impaired lexical activation or integration difficulties in PWA (e.g., Yee et al. 2008; Ferrill et al. 2012). Impaired short‐term verbal memory in PWA (see, e.g., Martin et al. 2018) may also reduce the lexical boost in PWA, especially if, as several accounts speculate (e.g., Traxler et al. 2014; Tooley 2020; Chang et al. 2012) the lexical boost relies primarily on short‐lived activation of processed lexical items in short‐term memory. However, until future studies confirm the relationship between cognitive functioning and structural priming, this conclusion will remain speculative. Our findings suggest that it is abstract syntactic priming that is crucial for creating measurable changes across sentence production and comprehension processes, rather than priming of lexical‐specific information. Thus, while our hypothesis of stronger generalization following same‐verb training was not confirmed, future efforts to identify structural priming training parameters which lead to such generalization will remain worthwhile.

## 5 Conclusions

In all, this study demonstrated cross–modality treatment generalization from production to comprehension in aphasia using structural priming, an implicit paradigm that taps syntactic representations. Specifically, participants demonstrated faster response times and increased fixations to target pictures during sentence comprehension, although improvements in comprehension accuracy were not reliable. Notably, PWA and controls rely on distinct training mechanisms: while controls benefited from lexical overlap between primes and targets, PWA did not, and instead appeared to rely solely on the syntactic representational level. We make the case for the adaptation of structural priming to a formal treatment protocol in aphasia to effectuate sentence production as well as (some) sentence comprehension improvements using one implicit treatment.

## Conflicts of Interest

The authors have no conflicts of interest to disclose.

## Data Availability

All data associated with this manuscript are available on the Open Science Framework at https://osf.io/rhbue/.
